# Case report: Characterization of the immunologic and molecular landscape in a unique presentation of invasive lobular carcinoma with concurrent uterine carcinosarcoma treated with immunotherapy

**DOI:** 10.3389/fimmu.2024.1422342

**Published:** 2024-07-15

**Authors:** Courtney J. Riedinger, Caprice D. Eisele, Ashwini Esnakula, Daniel G. Stover, Aharon G. Freud, Casey M. Cosgrove

**Affiliations:** ^1^ Department of Obstetrics and Gynecology, Division of Gynecologic Oncology, The Ohio State University Comprehensive Cancer Center/James Cancer Hospital, Columbus, OH, United States; ^2^ The Ohio State University, Columbus, OH, United States; ^3^ Department of Pathology, The Ohio State University Wexner Medical Center, Columbus, OH, United States; ^4^ Department of Internal Medicine, Division of Medical Oncology, The Ohio State University Comprehensive Cancer Center/James Cancer Hospital, Columbus, OH, United States

**Keywords:** carcinosarcoma, lobular breast cancer, epithelial to mesenchymal (EMT), immunotherapy, NK cell (NKC)

## Abstract

Invasive lobular breast cancer (ILC) is characterized by a relatively high risk for late recurrence and a unique metastatic pattern with an increased risk for metastasis to gynecologic organs and peritoneum. We present a unique case of recurrent ILC with metastasis to the abdominal peritoneum as well as the uterine myometrium and cervix. Treatment was complicated by the discovery of concomitant uterine carcinosarcoma. This patient was effectively treated with a combination of hormonal therapy for her metastatic ILC and a combination of chemotherapy and immunotherapy for uterine carcinosarcoma. Molecular evaluation revealed a characteristic *CDH1* mutation within the ILC and a *PI3KCA* mutation within the uterine carcinosarcoma, both of which have been linked to epithelial-to-mesenchymal transitions. Examination of the tumor immune microenvironment revealed proportionally more cytotoxic NK cells. This robust immune infiltration may be an indicator of the response to immunotherapy observed in this tumor or a result of the metastatic breast cancer within the uterus. This report provides a characterization of the molecular and immunologic landscape in this case with metastatic ILC and uterine carcinosarcoma.

## Introduction

Invasive lobular breast cancer (ILC) accounts for 5–15% of breast cancer cases (1). Loss of E-cadherin expression via the CDH1 gene is a hallmark of ILC and has been associated with epithelial-to-mesenchymal transition (2). ILC has a relatively high risk for late recurrence (3) with an increased frequency of involvement of bone, gastrointestinal sites, gynecologic organs, and peritoneum (4, 5). When breast cancer metastasizes to gynecologic organs, the ovaries are the most frequent metastatic site, and the uterus and cervix are rarely involved (6–8). We report a rare case of lobular breast cancer metastatic to the uterus and cervix which was discoveredwith concomitant uterine carcinosarcoma. An analysis of the tumor immune microenvironment and mutational profile of each component is presented.

## Results

### Case report

**Figure 1:**
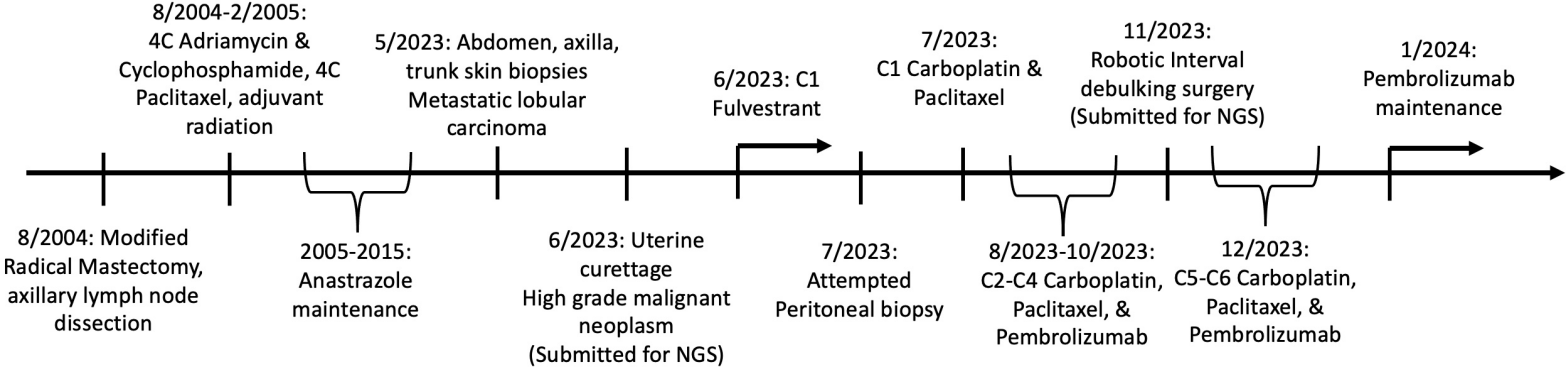
Timeline of patient’s oncologic care and collection of specimens for pathologic and molecular evaluation.

An 86-year-old woman, with a remote history of breast cancer [T2N3 estrogen receptor (ER)/progesterone receptor (PR) positive invasive lobular carcinoma] 10 years prior was treated with mastectomy and adjuvant chemotherapy, followed by anastrozole. The patient was noted to have skin lesions which were consistent with ILC recurrence on biopsy. The patient was started on fulvestrant for treatment of recurrent ILC. The patient reported post-menopausal bleeding and was referred to gynecologic oncology. Computerized tomographic imaging was concerning for carcinomatosis, a heterogeneous uterine cavity, right adnexal mass, and right axillary lymphadenopathy. The patient underwent an exam under anesthesia with vaginal biopsies and curettage with notable bulky pelvic disease. Pathology revealed a biphasic high-grade malignancy with epithelial and mesenchymal differentiation (**Figure 1A**) consistent with uterine carcinosarcoma with cervical involvement. An attempt was made to biopsy the omental nodularity given the uncertainty in diagnosis (metastatic breast cancer versus high-grade endometrial cancer), but this was unsuccessful, and given bulky pelvic disease we opted to proceed with neoadjuvant chemotherapy. The patient was started on platinum-based doublet with pembrolizumab (based on her elevated tumor mutational burden (TMB) and data from RUBY (9) and GY018 (10) clinical trials) for presumed high-grade endometrial cancer (EC) and was continued on fulvestrant therapy for her recurrent ILC. She had resolution of skin lesions after 4 months of hormonal therapy and four cycles of chemotherapy and imaging showed a decrease in axillary lymphadenopathy and decreased carcinomatosis. She underwent minimally invasive interval debulking. Pathology from interval debulking revealed uterine carcinosarcoma within the endometrium and involving cervical stroma as well as metastatic ILC involving the uterus, cervix, bilateral adnexa, omentum, and peritoneal implants. Following two additional cycles of chemotherapy and pembrolizumab, she was transitioned to pembrolizumab maintenance and continued on fulvestrant. Imaging and exam at the completion of chemotherapy showed excellent response to chemotherapy, immunotherapy, and hormonal therapy with no evidence of disease. A timeline of the patient’s oncologic care and the collection of specimens for pathologic and molecular evaluation is presented in **Figure 1**.

### Histopathology

**Figure 2:**
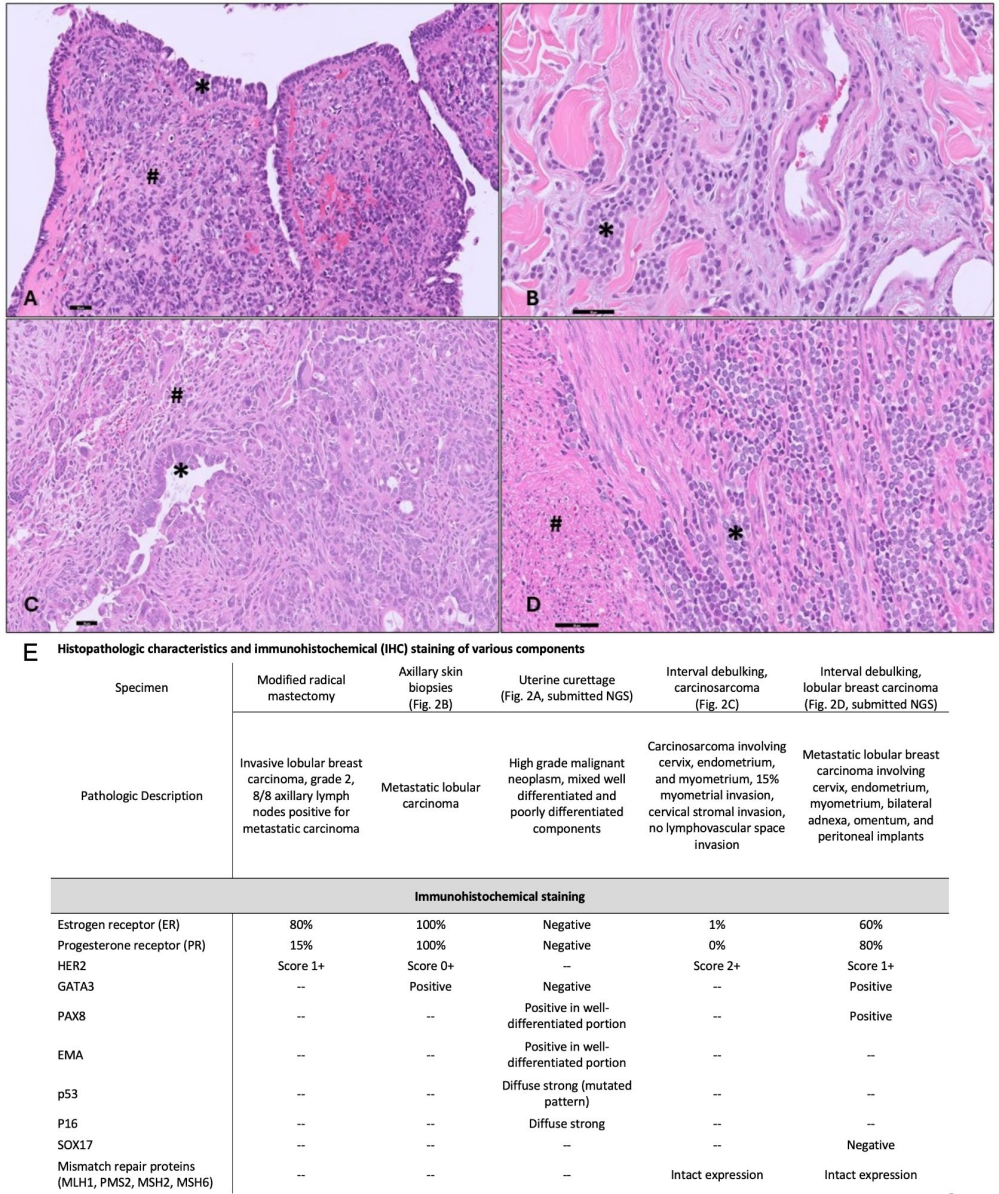
Histopathologic Data. **(A)** Uterine curettage with biphasic high-grade neoplasm with mixed high-grade carcinoma (*) and associated undifferentiated sarcomatous component (#) (200X). **(B)** Skin biopsies with classic lobular carcinoma with discohesive, small monomorphic tumor cells infiltrating the dermis (*) (400X). **(C)** Uterine tumor with high-grade carcinoma component (*) and sarcomatous component with spindled and rhabdoid morphology(#) (200X). **(D)** Metastatic lobular carcinoma (*) within the uterine myometrium (#) (400X). (scale bar=50 microns). **(E)** Summary of histopathologic and immunohistochemical (IHC) staining of various pathologic specimens. Abbreviations: Epithelial membrane antigen (EMA), Test not performed (–).

Pathology from the exam under anesthesia at diagnosis revealed biphasic high-grade neoplasm with mixed high-grade epithelial and associated poorly differentiated components showing epithelioid and spindle cell morphology (**Figure 2A**). Both components show diffuse expression for p16 and aberrant mutation-type expression for p53 (negative for GATA3, ER/PR) (**Figure 2E**).

We performed a secondary evaluation of the skin biopsies which demonstrated features of classic lobular carcinoma with discohesive, small monomorphic tumor cells infiltrating the dermis (**Figure 2B**). The tumor cells showed diffuse expression for GATA3, ER/PR (**Figure 2E**), supporting the diagnosis of metastatic ILC. Pathology from the interval debulking (collected after 5 months of fulvestrant and 4 cycles of neoadjuvant carboplatin and paclitaxel, 3 cycles of pembrolizumab) revealed a mass within the uterus which demonstrated a biphasic neoplasm composed of a high-grade epithelial glandular component and a sarcomatous component consisting of epithelioid to spindled high-grade tumor cells. Frequent stromal hyalinization with chronic inflammation and clusters of maturing rhabdoid tumor cells were consistent with partial tumor regression (**Figure 2C**). The tumor was myoinvasive with endocervical stromal invasion. In addition, foci of metastatic lobular carcinoma were noted in the myometrium (**Figure 2D**), endocervix, bilateral adnexa, omentum, and peritoneal implants.

### Molecular and immunologic evaluation

Next-generation sequencing (NGS) was performed upon a sample of the uterine carcinosarcoma from the uterine curettage prior to therapy and on the lobular breast cancer metastatic to the ovary which was collected at the time of interval debulking. Differences in molecular alterations were noted between tumor samples of the carcinosarcoma and lobular breast carcinoma (**Supplementary Table 1**). The lobular breast cancer metastatic to gynecologic organs demonstrated a pathogenic mutation in cadherin-1 (*CDH1*, c.476del, p.P159fs), which is a hallmark of ILC. In addition, a pathogenic mutation in *TET2* (c.5423_5424del, p.R1808fs) was noted, which functions as part of the WT1 pathway. In contrast, the uterine carcinosarcoma was found to have pathogenic mutations in *PIK3CA* (c.1132T>C, p.C378R), *TP53* (c.518T>G, p.V173G), *ARID1A* (c.2044_2047del, p.5682fs), *KRAS* (c.35G>C, p.G12A), with a copy number gain in *MYCN*.

**Figure 3:**
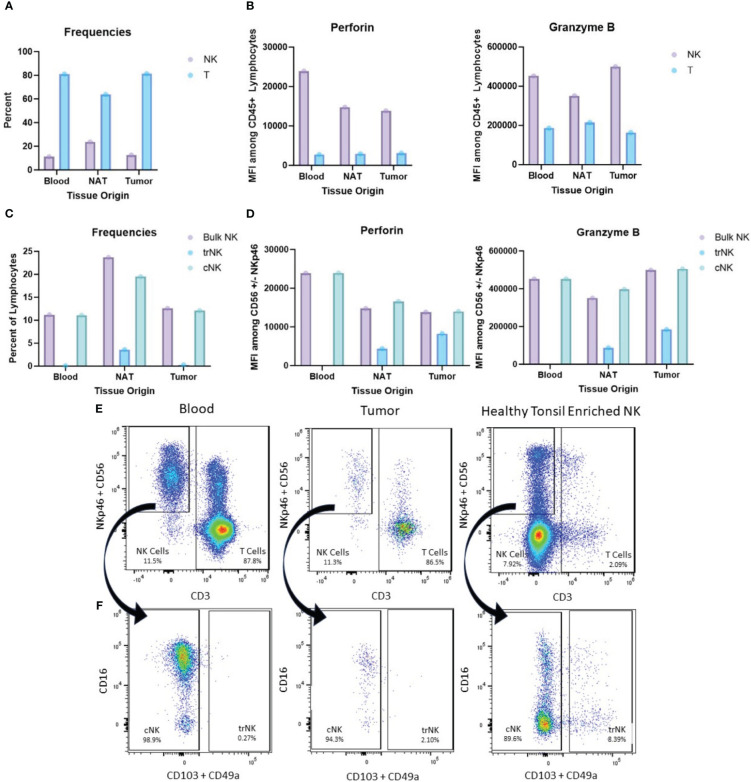
Immune cell phenotyping by flow cytometry. **(A)** Proportion of NK (Live, Lineage-CD56+NKp46+) cells and T (Live CD3+) cells in patient blood, normal uninvolved adjacent tissue (NAT), and uterine tumor. **(B)** Mean Fluorescence Intensity (MFI) of Perforin and Granzyme B by intracellular flow cytometry among three patient tissue origins. **(C)** Proportion of tissue-resident NK cells (trNK) (Live, Lineage-CD56+NKp46+CD103+) and cytotoxic NK cells (cNK) (Live, Lineage-CD56+NKp46+CD103-) cells among three patient tissue origins. **(D)** MFI of Perforin and Granzyme B by intracellular flow cytometry of trNK and cNK cells among three patient tissue origins. **(E)** Flow cytometry gated for NKp46+, CD56+, and CD3+ cells demonstrating proportion of NK and T cells with tonsil used for control. **(F)** Flow cytometry gated for CD16+, CD103+, and CD49a+ cells demonstrating cNK and trNK with tonsil used for control.

Among variants of unknown significance (VUS), two were identified in both carcinosarcoma and ILC samples with approximately 50% VAF, including *NOTCH1* (c.6685G>A, p.V229M, VAF 60.6% carcinosarcoma, 48.1% lobular) and *TSC1* (c.2647G>A, p.A883T, VAF 58.5% carcinosarcoma, 40.9% lobular). Additionally, both tumors showed a shared *KMT2C* mutation (c.2521C>T, p.R841W) although with lower VAF, 14.5% in the lobular breast cancer and 8.1% in the uterine carcinosarcoma. The TMB of the carcinosarcoma component was 10.0 mutations/MB compared to 5.3 mutations/MB in the metastatic lobular breast cancer. The patient underwent germline genetic testing without pathogenic mutation. Additionally, we performed a phenotypic analysis of the tumor immune microenvironment (TIME) of the uterine tumor for research purposes with a focus on T cell and NK cell phenotype and cytolytic potential. While T cells were the most abundant immune cells in all three samples (**Figures 3A, E**), the NK cells within the uterine tumor had proportionally higher expression of perforin and granzyme B (**Figure 3B**). Ongoing work from our lab (11) and others (12, 13) suggests a dichotomy among NK cell populations, with one subset expressing tissue-resident NK cell (trNK) markers such as CD103 and CD49a, whereas the other subset lacks these markers and instead expresses high levels of CD16 and is akin to conventional cytotoxic NK cells (cNK) found in circulation. Degos et al. performed an evaluation of previously untreated endometrial cancers and demonstrated that chemokines and cytokines within the tumor microenvironment may inhibit the recruitment and cytotoxic function of NK cells within the tumor (14). Degos reported that the NK cells were rare within endometrial cancers and the majority of NK cells within endometrial tumors expressed inhibitor molecules to block cytotoxic function (14). Our lab has also identified a paucity of cNK cells within endometrial cancers, particularly within type 2 endometrial cancers which typically have more aggressive behavior (11). While this case of endometrial cancer with carcinosarcoma histology would be classified as a type 2 endometrial cancer, her tumor displayed an overwhelming proportion of cNK cells (**Figures 3C, D, F**). The striking presence of functional granzyme B+ perforin+ cNK cells in this tumor (**Figure 3D**) suggests an active TIME comprised of largely responsive NK cells.

## Discussion

Invasive lobular carcinoma is the second most common histologic variant of breast cancer. Classic ILC demonstrates small round discohesive cells that grow in linear strands through the surrounding stroma and may form concentric rings around the ducts. ILC is frequently ER/PR positive and HER2 neu negative tends to be multicentric and occurs in older women (15, 16). Loss of cell-cell adhesion molecules has been identified as a phenotypic driver for ILC with genetic or epigenetic *CDH1* loss in more than 90% of cases (2, 17). The Cancer Genome Atlas has also documented loss of *PTEN*, activation of *AKT*, and mutations in *TBX3* and *FOXA1* (18).While ILC is recognized for an increased risk for late recurrence and peritoneal spread, the metastatic pattern of this case is interesting to discuss. In addition to late recurrence, approximately 10 years from primary treatment, she demonstrated skin metastasis, peritoneal involvement, and diffuse involvement of the uterus and cervix. Pathology was interesting in that the ovaries, uterine myometrium, and cervix demonstrated histopathology consistent with the patient’s recurrent ILC which was similar to metastatic skin lesions. Within the uterus and invading the myometrium and cervix we also found tumor consistent with uterine carcinosarcoma. While prior literature supports gynecologic organs as a site of metastasis for lobular breast carcinoma, the ovaries are much more frequently involved, and the uterus and cervix are rarely involved (6–8). To our knowledge, this is the first case of lobular carcinoma co-existing with carcinosarcoma in the uterus and cervix. Uterine carcinosarcoma is a biphasic entity with carcinomatous (epithelial) and sarcomatous (mesenchymal) components (17, 19). These biphasic tumors are monoclonal in origin with the carcinoma component secondarily undergoing metaplasia into the sarcomatous component (19–21). Theoretically, during the 10-year period between primary diagnosis and recurrence, the loss of E-cadherin expression within the lobular breast carcinoma may have enabled an epithelial-to-mesenchymal transition that could allow dedifferentiation to a clonal population capable of developing into carcinosarcoma. However, NGS in this case revealed the hallmark loss of E-cadherin (*CDH1*, c.476del) within the lobular breast carcinoma (metastatic to the ovary) which was not identified within the uterine carcinosarcoma which makes it difficult to consider loss of *CDH1* as a driver mutation for both tumors. The uterine carcinosarcoma did demonstrate a *PI3KCA* (c.1132T>C) mutation which has also been implicated in epithelial-to-mesenchymal transition (22). While the ILC and carcinosarcoma were noted to share mutations in *NOTCH1* (c.6685G>A) and *TSC1* (c.2647G>A) we were unable to send paired normal samples and it is possible that these VUS could be germline mutations explaining the expression within both tumors. While it is possible the recurrent invasive lobular breast carcinoma underwent sarcomatous dedifferentiation to exhibit the high-grade epithelial and sarcomatous components of the uterine carcinosarcoma the authors feel that this is less likely. Another possibility is that this is a collision tumor with the metastatic lobular carcinoma colliding with a uterine carcinosarcoma and creating a tumor micro-environment where both could thrive. While both tumors demonstrate *NOTCH1* and *TSC1* mutations the two tumors do not share *CDH1* or *PIK3CA* mutations which could serve as driver mutations of epithelial-to-mesenchymal transition.

Our evaluation of the immune landscape is also noteworthy. Contrary to prior research (11–14), this tumor, after exposure to checkpoint blockade, demonstrated proportionately more cytotoxic NK cells and fewer quiescent tissue-resident NK cells. This immune infiltrate may be a marker of response to the combination of chemotherapy and checkpoint blockade. PD-1 expression on NK cells has been associated with impaired proliferation and cytolytic activity in ovarian and lung cancer patients (23, 24) and preclinical studies have demonstrated an increase in NK cell-mediated cytolytic activity in response to PD-L1 blockade (25, 26). It is also possible that the presence of breast cancer metastasis within the uterus primed the environment for a robust immune infiltrate.

The management of this patient’s concurrent invasive lobular carcinoma and uterine carcinosarcoma presented a therapeutic dilemma. Given the patient’s hormone receptor status, fulvestrant was initiated for the treatment of her metastatic breast cancer. Although CDK4/6 inhibition was considered, it was deferred in the setting of the patient’s age, advanced-stage endometrial cancer, and concern about potential drug tolerance in combination with chemotherapy and immunotherapy. Neoadjuvant chemotherapy with platinum doublet and pembrolizumab was chosen due to the patient’s bulky pelvic disease, high-grade endometrial cancer, and elevated TMB. Notably, the uterine carcinosarcoma lacked estrogen or progesterone receptors, suggesting limited benefit from the fulvestrant administered for metastatic breast cancer. While platinum-based chemotherapy and immunotherapy are occasionally employed in metastatic breast cancer, they typically do not constitute the first-line treatment for hormone receptor-positive breast cancer. The patient was noted to have a quick resolution of skin metastatic deposits and axillary lymphadenopathy with the combined treatment approach of fulvestrant, platinum-based chemotherapy, and immunotherapy. Pathological assessment of the interval debulking specimen further confirmed an excellent treatment response. Importantly, this multifaceted treatment regimen was well tolerated by our elderly patient.

In conclusion, metastatic lobular breast cancer may present atypically and involve gynecologic structures. The possibility of divergent histopathology must be considered, and tissue biopsy may enable molecular evaluation to guide therapy. With the increasing utilization of biomarker-driven therapy in EC (27) the critical examination of molecular testing in each of these disease entities will be key to precision therapy.

## Methods

The patient provided informed consent for publication of this manuscript and informed consent is maintained on file at The Ohio State University. The patient signed informed consent for molecular and immunologic testing under a study approved by the Institutional Review Board (IRB#2020C0066). All authors have complied with all relevant ethical regulations including the Declaration of Helsinki.

### Molecular and immunologic evaluation

A multigene panel (648 genes) was used to evaluate for oncologic driver mutations in representative samples (Tempus XT panel, Tempus, Chicago, IL, USA). Tumor mutational burden was estimated (28). A phenotypic analysis of the tumor immune microenvironment (TIME) using ex-vivo flow cytometry of fresh uterine tumor, alongside autologous blood, and normal uninvolved uterine tissue. The samples were dissociated to a single cell suspension and stained for various immune cell (CD45+) markers including Natural Killer (NK) cells (CD56, NKp46+) and T Cells (CD3+), along with effector molecules (Granzyme B, PD-1, and Perforin). Healthy pediatric tonsil which was enriched for NK cells was used as a staining control.

## Data availability statement

The raw data supporting the conclusions of this article will be made available by the authors, without undue reservation.

## Ethics statement

The studies involving humans were approved by The Ohio State University James Cancer Hospital. The studies were conducted in accordance with the local legislation and institutional requirements. The participants provided their written informed consent to participate in this study. Written informed consent was obtained from the individual(s) for the publication of any potentially identifiable images or data included in this article. Written informed consent was obtained from the participant/patient(s) for the publication of this case report.

## Author contributions

CR: Conceptualization, Formal analysis, Investigation, Methodology, Resources, Writing – original draft, Writing – review & editing. CE: Data curation, Formal analysis, Investigation, Methodology, Resources, Writing – original draft, Writing – review & editing. AE: Conceptualization, Data curation, Formal analysis, Investigation, Methodology, Resources, Visualization, Writing – review & editing. DS: Conceptualization, Investigation, Resources, Supervision, Validation, Writing – review & editing. AF: Conceptualization, Data curation, Formal analysis, Investigation, Methodology, Project administration, Resources, Supervision, Visualization, Writing – review & editing. CC: Conceptualization, Data curation, Formal analysis, Funding acquisition, Investigation, Methodology, Project administration, Resources, Supervision, Validation, Visualization, Writing – review & editing.
