# Supplementary material for: Case report: Characterization of the immunologic and molecular landscape in a unique presentation of invasive lobular carcinoma with concurrent uterine carcinosarcoma treated with immunotherapy
Source: Front Immunol. 2024 Jul 15;15:1422342. doi: 10.3389/fimmu.2024.1422342 (PMC11284112; doi:10.3389/fimmu.2024.1422342)
Supplement: Supplementary file 1 [file Table_1.docx]

| **Supplementary Table 1: Comparison of Next-Generation Sequencing (NGS) findings** | | |
| --- | --- | --- |
| Specimen | Uterine curettage (untreated)  Tumor percentage 60% | Interval debulking, sample sent from ovary with metastatic lobular breast carcinoma  Tumor percentage 10% |
| Pathogenic mutations | *PIK3CA*, c.1132T>C, p.C378R | *CDH1*, c.476del, p.P159fs |
|  | (VAF 46.7%) | (VAF 4.4%) |
|  | *TP53*, c.518T>G, p.V173G | *TET2*, c.5423_5424del, p.R1808fs |
|  | (VAF 78.3%) | (VAF 5.5%) |
|  | *ARID1A*, c.2044_2047del, p.5682fs |  |
|  | (VAF 46.7%) |  |
|  | *KRAS*, c.35G>C, p.G12A |  |
|  | (VAF 22.9%) |  |
|  | *MYCN* copy number gain |  |
| Variants of Unknown Significance | NOTCH1, c.6685G>A, p.V2229M | NOTCH1, c.6685G>A, p.V2229M |
|  | (VAF 60.6%) | (VAF 48.1%) |
|  | TSC1, c.2647G>A, p.A883T | TSC1, c.2647G>A, p.A883T |
|  | (VAF 58.5%) | (VAF 40.9%) |
|  | KMT2C, c.2521C>T, p.R841W | KMT2C, c.2521C>T, p.R841W |
|  | (VAF 8.1%) | (VAF 14.5%) |
|  | SYNE1, c.25199G>A, p.S58400N | G6PD, c.940G>A, p.V314I |
|  | (VAF 62.0%) | (VAF 6.3%) |
|  | CHD4, c.2981T>C, p.F994S | IL7R, c.37T>C, p.S13P |
|  | (VAF 56.0%) | (VAF 5.2%) |
|  | WNK2, c.199C>T, p.P67S | CUL4A, c.17C>T, p.6L |
|  | (VAF 55.2%) | (VAF 5.1%) |
|  | RECQL4, c.1868G>A, p.R623H | NCOR1, c.3652C>T, p.H1218Y |
|  | (VAF 54.6%) | (VAF 5.0%) |
|  | FANCM, c.2382A>G, p.I795V |  |
|  | (VAF 40.1%) |  |
|  | KMT2B, c.1444C>T, p.R482W |  |
|  | (VAF 37.5%) |  |
|  | IL10RA, c.320G>A, p.R107Q |  |
|  | (VAF 23.4%) |  |
|  | GEN1, c.2519_2523del, p.L840fs |  |
|  | (VAF 10.6%) |  |
| Tumor mutational burden (mutations/Megabase) | 10.0 mut/Mb | 5.3 mut/Mb |
